# Actual and Perceived Motor Competence in Chilean Schoolchildren before and after COVID-19 Lockdowns: A Cohort Comparison

**DOI:** 10.3390/bs13040306

**Published:** 2023-04-04

**Authors:** Jaime Carcamo-Oyarzun, Sonia Salvo-Garrido, Isaac Estevan

**Affiliations:** 1 CIAM Physical Literacy Research Centre, Faculty of Education, Social Science & Humanities, Universidad de La Frontera, Temuco 4780000, Chile; 2 Department of Physical Education, Sports and Recreation, Universidad de La Frontera, Temuco 4780000, Chile; 3 Department of Mathematics and Statistics, Universidad de La Frontera, Temuco 4780000, Chile; 4 Activitat Física i Promoció de la Salut (AFIPS) Research Group, Department of Teaching of Physical Education, Arts and Music, University of Valencia, 46022 Valencia, Spain

**Keywords:** COVID-19, fundamental motor skills, self-perception, cohort, childhood, physical education, motor development

## Abstract

(1) Background: the measures applied in Chile to reduce COVID-19 infections have been very strict, mainly for children who have experienced lockdowns and school closures for almost two years. Emerging evidence indicates that lockdowns have had several negative effects on children; therefore, the present study seeks to analyze the secular effects of COVID-19 lockdowns on Chilean students’ actual motor competence (AMC) and perceived motor competence (PMC). (2) Methods: using a sequential cohort design, data from 523 fifth-grade students at nine elementary schools (46.8% girls, age M = 11.11, SD = 0.66) were assessed in 2018–19 (pre-lockdown) (*n* = 265) and 2022 (post-lockdown) (*n* = 258). (3) Results: in the domain of object control (AMC and PMC), no significant differences were found (AMC *p* = 0.559; PMC *p* = 0.682). In the self-movement domain of AMC and PMC, the significant differences found presented a small effect size (AMC *p* = 0.044, *η_p_*^2^ = 0.01; PMC *p* = 0.001, *η_p_*^2^ = 0.03). (4) Conclusions: although the differences encountered were not drastic, self-movement skills tended to be greatly affected by the lockdowns resulting from the COVID-19 pandemic. These findings broaden information on the negative consequences of the pandemic on students in aspects related to an active and healthy life.

## 1. Introduction

Regular physical activity is recognized as one of the factors that most impact people’s quality of life [1]. For that reason, physical activity promotion and encouragement have generated great interest in recent decades, mainly because, despite the multiple benefits it produces, the proportion of people who are physically active is very low [2]. High levels of physical inactivity in children are concerning, given that a high global percentage of children and adolescents do not fulfill the recommendations of physical activity for their age [3], which negatively affects their health [4]. This worrisome situation has been amplified further by the health emergency resulting from the COVID-19 pandemic, which led to various negative consequences for children, such as increasing their level of physical inactivity, increasing their screen time, and worsening their sleep quality [5,6,7,8]. All of this is a product of the restrictive measures imposed by health authorities to deal with COVID-19 [9].

COVID-19 was declared a worldwide pandemic by the World Health Organization in March 2020 [10], causing a health emergency that produced various changes in people’s lives. To mitigate the rapid propagation of COVID-19 in the initial stages of the pandemic, government and health authorities in many countries took severe public health measures which significantly curtailed the day-to-day lives of these countries’ populations. One of the measures that directly affected people was the application of lockdowns which involved drastic restrictions in some countries, such as mandatory school closures [9,11]. To not interrupt learning processes, schools produced strategies to continue with classes, and distance education via the Internet was the predominately used methodology [12]. However, this situation intensified the problem of physical inactivity since the lockdowns caused children and adolescents to spend more time in front of screens, which reduced physical activity compared to periods before the lockdowns [5]. In addition, school closures meant that students had limited access to physical activity during school hours because of a lack of physical education classes, breaks, and travel to and from school [8]. Thus, it is feared that although the COVID-19 pandemic is under control, it may continue affecting the population in various ways; for example, physical inactivity may have long-term consequences regarding people’s physical and mental health [4,13,14].

The promotion of active lifestyles during and after the pandemic in school populations is one of the main challenges to pursue to counteract the prevalence of sedentary behaviors [4]. It is, therefore, necessary to understand how factors associated with the habitual practice of physical and sports activities have been affected by the pandemic. One of these factors is actual motor competence (AMC), which is considered a relevant factor in the initiation, continuation, or abandonment of the practice of physical activity [15,16,17]. AMC can be defined as a person’s ability to execute basic motor skills, particularly during childhood [18]. These motor skills have been grouped as locomotor skills, object control, and stability [19]. Thus, being competent in a variety of motor skills is a requirement for the performance of daily activities and participation in health-related physical activity during childhood, adolescence, and adulthood [20]. Adequate levels of AMC are required to participate in various physical activities, such as active games and sports, because they improve self-regulation mechanisms, including expectations of self-efficacy and intrinsic motivation [21]. It has thus been conceptualized that the reciprocal and dynamic relations between AMC and the practice of physical activity throughout childhood are mediated by factors such as the perception of motor competence and health-related physical conditions [20,22,23]. The perception of competence or perceived motor competence (PMC) is understood as a person’s belief in their own ability to perform certain motor skills [20], or in other words, AMC-related self-perception [24]. Emerging evidence indicates that PMC interacts with AMC and generates important underlying mechanisms that influence adherence and persistence in the practice of physical activity [25,26]. Individuals with high levels of PMC are more willing to participate in physical activities and more likely to persist in tasks that may be perceived as challenging than those with low levels of PMC [20,27]. A child’s PMC is important for their well-being, social acceptance, participation in active games, and their willingness to participate in physical education classes and physical activities in general [24]. Hence, PMC can be an important predictor of active lifestyles [21], given that when children do not think they are competent in a task, they will likely choose not to participate in it [20].

As with children’s physical activity levels, evidence is emerging that AMC has also been negatively affected by the COVID-19 pandemic. Studies that addressed the topic from a longitudinal perspective [28] and a sequential cohort perspective [29] concluded that the lockdowns resulting from the pandemic led to reduced motor performances in children. In terms of PMC, there are few studies on its development during the pandemic, although there is evidence that after a year of lockdown, and despite not having had physical education classes in person, the PMC of Chilean students remained stable [30]. Considering the important connections between AMC and PMC and physical activity and the ceasing of lockdown measures to fight the COVID-19 pandemic, it is necessary to determine whether AMC and PMC have been affected after almost two years of school closures. For that reason, the present study seeks to compare the AMC and PMC of fifth-grade elementary Chilean students from a pre-lockdown cohort (2018–2019) and a post-lockdown cohort (2022).

## 2. Materials and Methods

### 2.1. Participants

As this study uses a sequential cohort design, two cohorts have been included: the pre-lockdown cohort corresponds to Fondecyt project 11170525, conducted in 2018–2019, while the post-lockdown cohort corresponds to Fondecyt project 1210616, started in 2022. Both studies have the approval of the Scientific Ethics Committee of the Universidad de La Frontera (file No. 125_17 and No. 040_21, respectively).

For the entire analysis, data from 523 fifth-grade elementary students (46.8% girls, age M = 11.11 SD = 0.66) in nine schools in Temuco, Chile, were assessed. Of these 523 students, 265 are from the pre-lockdown cohort (43.0% girls, age M = 11.6 SD = 0.56), and 258 from the post-lockdown cohort (40.3% girls, age M = 11.5 SD = 0.49). The schools to which the pre-lockdown cohort students belong (4 schools) and those in the post-lockdown cohort (5 schools) had similar characteristics in the two projects. All the schools are considered highly vulnerable according to the Chilean government’s National School and Scholarship Assistance Council (JUNAEB in Spanish) [31]. All the participants had an informed consent signed by their parents or guardians, and the children who participated signed an informed consent declaring their voluntary participation in the study.

### 2.2. Instruments

#### 2.2.1. Assessment of AMC

The MOBAK 5-6 (Motorische Basiskompetenzen in German) was used, created by Herrmann and Seelig [32], and validated in Spanish for the Chilean school population [33,34]. This test evaluates the AMC of elementary school students in fifth and sixth grade (10–12 years). It consists of eight motor tasks (items) organized in two domains: (a) object control, composed of test items for handling a ball (catching, throwing, bouncing, and dribbling); and (b) self-movement, composed of test items for body coordination in space (balancing, jumping, running and rolling). A product-oriented test is included, although the evaluation focuses on functionality through the resolution of motor tasks. Thus, each item scores from zero to two points depending on the number of successes, and then they are all added together to provide a score for each dimension. Thus, the score range in each domain goes from a minimum of zero points to a maximum of eight points. The validity of the MOBAK 5-6 for Chilean schoolchildren showed a satisfactory model fit (*χ*^2^ = 55.48; *df* = 19; *p* = 0.001; CFI = 0.926; RMSEA = 0.051) [34].

#### 2.2.2. Assessment of PMC

The SEMOK questionnaire (Selbstwahrnehmung der motorischen Kompetenz in German) by Herrmann and Seelig [35] was used and validated in Spanish by Carcamo-Oyarzun et al. [36]. It consists of eight items aligned with the MOBAK 5-6 test, where students must indicate the extent to which they consider themselves able to carry out the eight motor tasks of the MOBAK 5-6 test, answering the question, “Do you think you can do the following activities?” The answer format is a Likert scale from one to five (1 = totally disagree, 5 = totally agree), where the students indicate their degree of agreement with the statement in each item. In addition to the verbal description, all the items are accompanied by a graphical representation of the motor task to reinforce their understanding. For this study, the internal consistency of SEMOK was acceptable/good (α = 0.78 overall; α = 0.78 object control; α = 0.66 self-movement). Furthermore, a confirmatory factor analysis of the relationships between AMC and PMC in Chilean schoolchildren showed an acceptable adjustment index (*χ^2^* = 192.81; *df* = 98; *p* < 0.001; CFI = 0.90; RMSEA = 0.05) [36].

#### 2.2.3. Co-Variable Body Mass Index (BMI)

BMI was determined through the formula kg/m^2^. Weight was determined using a calibrated scale, and height was measured using a stadiometer (SECA, Hamburg, Germany), asking participants to take the measurements barefoot and with as few clothes as possible.

### 2.3. Procedures

The students in the pre-lockdown cohort were evaluated between August 2018 and October 2019 as part of Fondecyt project 11170525, whereas the data collection of the post-lockdown cohort took place between April and June 2022 as part of Fondecyt project 1210616. For both cohorts, the data collection procedure was the same, following the protocols established in the first project [34]. Most of the assessment group members were trained in the application of the MOBAK test and participated in both projects.

The evaluations took place during physical education classes, where an evaluator met with the students in their classroom to present and explain the SEMOK questionnaire and how to respond. The estimated time to answer the questionnaire was between 10 and 15 min. Then, the students went to the gymnasium, where eight evaluators took the measurements. Each evaluator was responsible for and accompanied a group of three to five children, who went through each station until all the tasks had been completed, including the weight and height evaluation. At each station, the evaluator explained the motor task and then demonstrated it. Following the test protocol, each child carried out the tasks without allowing any attempts first. The approximate duration of the complete application of the evaluation was between 45 and 60 min for each class group.

### 2.4. Description of the Lockdown Period

In 2020, the Chilean government made lockdown mandatory for the residents of Temuco in March [37], suspending schools almost as soon as the new school year had begun, and it stayed that way until July 2021. So as not to interrupt the education process during this period, various remote strategies were established, mainly via the Internet. The schools to which the students in the post-lockdown cohort belonged reported that, at the beginning of the pandemic, they were sent practical and theoretical work weekly via e-mail, which contained instructions to perform physical exercises and motor activities, in addition to recommendations for healthy living and eating according to the objectives prioritized by the Chilean Ministry of Education [38]. However, considering the length of the lockdown, they began to use online platforms for physical education classes. Given the students’ socioeconomic level and according to reports by the physical education teachers, the percentage of class participation was very low throughout that period [39].

From March to December 2021 (the second academic semester in Chile), there was a partial voluntary return to in-person classes. For this, schools adopted a hybrid model, where half the students attended in-person classes while the other half followed the classes online. Participation in this hybrid model was also intermittent, with periods where all students were at home participating in online classes due to some infections that occurred in their respective schools. Similarly to the period of classes prior to this hybrid model, in-person and online attendance was very low. Even in March 2022, when the obligatory return to classes was decreed in Chile, the attendance percentage in the first week was 68% [40], which increased to 77% in April 2022 [41], when the data collection for this study began.

### 2.5. Statistical Analysis

For the descriptive statistics, the measures of central tendency (mean and standard deviation) were calculated. To determine the normality of the sample, the standardized coefficients of skewness and kurtosis were used. Later, an analysis of variance (ANOVA) was applied to determine the differences in AMC (actual object control and actual self-movement) and PMC (perceived object control and perceived self-movement) between the pre-lockdown and post-lockdown cohorts. The analysis of the variables of AMC and PMC were adjusted for gender, age, and BMI. A level of significance was established at *p* < 0.05, and the effect size was determined through *η_p_^2^* (interpretation: values smaller than 0.01 trivial, between 0.01 and 0.06 small, between 0.06 and 0.14 moderate, and higher than 0.14 large) [42]. All the analyses were performed with the SPSS statistics package version 25.0 (IBM Corp., Armonk, NY, USA).

## 3. Results

**Figure 1:**
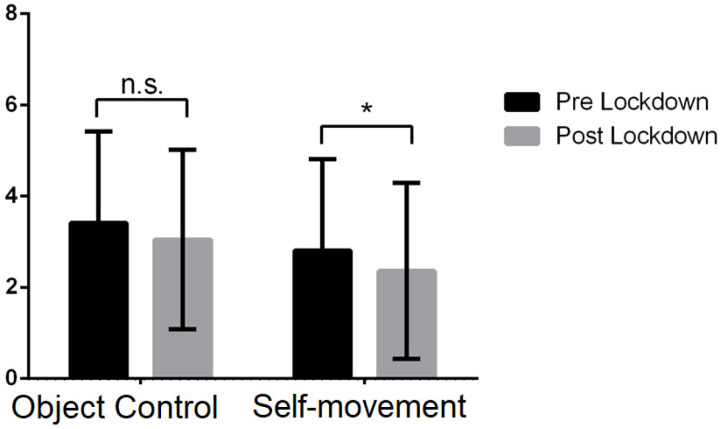
Comparison of AMC (object control and self-movement) pre- and post-lockdown. *n.s.* means no significant differences, whereas * means significant differences (*p* < 0.05).

Table 1 provides the descriptive analyses of the results from the pre-lockdown and post-lockdown cohorts. In terms of the AMC (Figure 1) in the domains of object control and self-movement, the students in the post-lockdown cohort exhibited similar values to the students in the pre-lockdown cohort. However, the analysis of variance of the domain of object control showed that these differences are not significant (*F*(1, 447) = 0.343, *p* = 0.559), where the effect of the co-variables BMI (*F*(1, 447) = 6.305, *p* = 0.012) and gender (*F*(1, 447) = 50.358, *p* < 0.001) was significant, but not age (*F*(1, 447) = 0.661, *p* = 0.417). The overall model explained 11.7% (*R*^2^ = 0.117) of the variance of AMC. For the domain of self-movement, the analysis of variance indicates that the pre-lockdown exhibited higher AMC than the post-lockdown cohort (*F*(1, 447) = 4.080, *p* = 0.044, *η_p_*^2^ = 0.01). The effect of the co-variables age (*F*(1, 447) = 12.099, *p* = 0.001) and BMI (*F*(1, 447) = 57.733, *p* < 0.001) was significant, but not gender (*F*(1, 447) = 3.394, *p* = 0.066). The overall model explained 16.0% (*R*^2^ = 0.160) of the variance.

**Figure 2:**
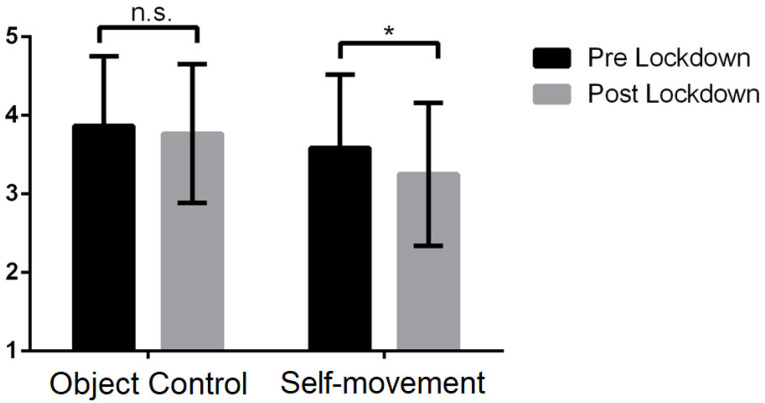
Comparison of PMC (object control and self-movement) pre- and post-lockdown. *n.s.* means no significant differences, whereas * means significant differences (*p* < 0.05).

With respect to the PMC (Figure 2), the students of the post-lockdown cohort present similar values in the PMC object control and lower values in PMC self-movement than the students in the pre-lockdown cohort. The analysis of variance shows that, in object control, these differences are not statistically significant (*F*(1, 447) = 0.317, *p* = 0.574), where the effect of the co-variables BMI (*F*(1, 447) = 5.610, *p* = 0.018) and gender (*F*(1, 447) = 11.725, *p* < 0.001) was significant, but not age (*F*(1, 447) = 0.191, *p* = 0.662). The overall model explained 4.1% (*R*^2^ = 0.41) of the variance. On the other hand, the analysis of variance corresponding to the self-movement domain showed statistically significant differences between the pre- and post-lockdown cohorts (*F*(1, 447) = 11.500, *p* = 0.001, *η_p_*^2^ = 0.025). The effect of the co-variables gender (*F*(1, 447) = 16.350, *p* < 0.001) and BMI (*F*(1, 447) = 38.257, *p* < 0.001) was significant, but not age (*F*(1, 447) = 3.132, *p* = 0.077). The overall model explained 14.5% (*R*^2^ = 0.145) of the variance.

## 4. Discussion

This study sought to analyze the secular effects of the COVID-19 lockdown in Chilean students’ AMC and PMC, that is, fifth-grade Chilean students in a pre-lockdown (2018–2019) and a post-lockdown (2022) cohort. The AMC-related results indicate that the students assessed post-lockdown tend to present a slightly poorer motor performance than those who attended the same grade before the COVID-19 pandemic began, specifically in self-movement skills. Although there are still few studies on the impact of COVID-19 on motor competence, this reduction in motor performance during the pandemic was also reported by Wessely et al. [29] in a sequential cohort study where cohorts were compared on timelines before (2016) and during the pandemic (2020, 2021), with higher BMI and lower motor performance values appearing in the latter group, especially in the children from an underprivileged socioeconomic background. In another study with pre- and post-lockdown assessments, but from a longitudinal perspective evaluating the same students, Pombo et al. [28] found that post-lockdown AMC was significantly lower than the performance before the lockdown in all the motor tests (except jumping sideways). In addition, they found differences in all motor competence domains, which differs partially from our study, where there were no significant differences in the domain of object control. It is possible that in Chile, where the lockdown has lasted intermittently for almost two years with no access to parks or schools, children have access to resources for play at home that involve object control skills and handling, which do not require much space compared to self-movement skills, which need more space. This has led to lower AMC in post-lockdown self-movement.

With regard to PMC, few studies have addressed the topic considering the lockdown restrictions imposed by the COVID-19 pandemic. Carcamo-Oyarzun et al. [30] analyzed how the PCM evolves when students have not had physical education classes in person because of school closures due to COVID-19. For this, they evaluated the PMC in lockdown conditions (2020), comparing the results with assessments made on the same students as before the pandemic (2019), with no significant differences. These results differ somewhat from those in the present study, where there were indeed differences in the self-movement PMC. In line with what was discovered in AMC, self-movement PMC has been affected by the lockdown because the children have not been able to run long distances or play actively with other children, which in turn has meant an increase in BMI, which could lead them to feel less competent in these skills post-lockdown.

The lower values in AMC and PMC in self-movement skills in the post-lockdown cohort support the hypothesis that the restrictive pandemic measures might have affected the students’ AMC and PMC. School closures meant eliminating opportunities for physical activity, like physical education classes, sports activities, and recreation [43]. The lockdowns imposed confined students in their homes and favored sedentary behaviors [5,7,8], reducing the opportunities for active play. In addition, the lockdown also made outdoor activities difficult [6], taking away multiple opportunities for physical activity. Considering the interactions between physical activity, AMC, and PMC [20,27], these restrictions of movement are unfavorable to children’s physical activity behaviors [44].

For the Chilean school population, the lockdowns imposed by the health authorities were quite restrictive since, for other groups (such as older adults or people with autism), there were temporary authorizations to be able to leave their houses on certain days of the week [45]; however, children were not initially considered for these authorizations. Considering that Chilean children reduced their physical activity, increased their screen time, and their sleep quality dropped during the pandemic [46], this study shows that this lockdown period also entailed increases in BMI related to a decrease in their AMC and PMC in self-movement.

In light of the relevance of AMC and PMC for the participation of students in physical activity and these aspects having a positive influence on physical activity levels throughout life, the results of this study underscore the need to take measures for the development of AMC and PMC in Chilean students, since the lockdown period and school closures have reinforced the alarming problem of physical inactivity that was looming before the health restrictions. Studies related to children’s physical activity in Chilean schoolchildren show an increase in sedentary behavior over the past few decades [47,48,49], and the pandemic has aggravated this situation. Pre-pandemic data report that less than 15% of Chilean students fulfill the international recommendations for physical activity [50,51]. Since this condition worsened due to the pandemic, strategies must be sought to counteract this problem and generate integral opportunities for students to do physical activity [43]. Promoting physical activity in schools, considering approaches that develop physical literacy, can help not only to improve the levels of AMC and PMC but also to increase social interaction and levels of moderate to vigorous physical activity [52], benefitting the children’s well-being.

The results should be interpreted in relation to the limitations of the study. Although this study offers important information as one of the first works to include secular pre- and post-lockdown trends for the analysis of AMC and PMC, it is important to emphasize that it is not without limitations. The fact that the data come from two different projects has not allowed longitudinal comparisons to be made, which would enable a better understanding of the evolution of AMC and PMC in this lockdown period. However, the schools of the participating students had similar characteristics in both projects. In addition, it must be pointed out that the information on the students’ physical activity before, during, and after the lockdown was unavailable. Although the schools were closed, they reported that physical education classes were taken online without providing further details on the activities undertaken in these classes. Factors such as the type of activities performed, the expertise of the teacher in remote classes, or the frequency of connection to physical education classes could have helped better understand the results concerning motor competence and how these might have affected the physical activity levels. On the other hand, it is essential to highlight that the data collection instruments have been validated for the Chilean school population [33,34]. The application of these instruments has been the same in both projects, not only in terms of the procedures but also in terms of the group of evaluators who participated in both projects, ensuring identical assessment procedures for the two samples.

## 5. Conclusions

The present study shows that AMC and PMC in fifth-grade elementary school students have suffered a decrease when the pre-pandemic (2018–2019) and post-lockdown (2022) cohorts are compared, particularly with regard to the self-movement-related motor tasks. These findings make it possible to broaden the information on the negative consequences the pandemic has had on students in aspects related to an active and healthy life, adding the decline in AMC and PMC to the reduction in physical activity levels, increase in BMI, increase in sedentary behaviors and sleep problems reported in other studies. This sum of detrimental conditions underscores the need to take measures (e.g., school interventions that foster the development of AMC and PMC) that can counteract these adverse effects on students’ health.

## Figures and Tables

**Table 1:** Descriptive statistics of the characteristics of the cohorts and the AMC and PMC in their dimensions of object control and self-movement.

	Pre-Lockdown Cohort				Post-Lockdown Cohort			
	Boys (N = 151)	Girls (N = 114)	Overall (N = 265)		Boys (N = 154)	Girls (N = 104)	Overall (N = 258)	
	M (SD)	M (SD)	M (SD)	95% CI	M (SD)	M (SD)	M (SD)	95% CI
Age	11.65 (0.57)	11.59 (0.55)	11.63 (0.56)	[11.55–11.70]	11.44 (0.50)	11.47 (0.49)	11.45 (0.49)	[11.39–11.52]
BMI	22.01 (4.38)	21.87 (3.88)	21.95 (4.15)	[21.46–22.55]	22.89 (4.10)	23.25 (4.33)	23.03 (4.19)	[22.37–23.49]
AMC ^1^								
Object control	4.03 (1.90)	2.57 (1.84)	3.40 (2.01)	[2.98–3.50]	3.53 (2.03)	2.33 (1.65)	3.05 (1.97)	[2.84–3.37]
Self-movement	2.62 (1.95)	3.06 (2.06)	2.81 (2.00)	[2.53–3.05]	2.30 (1.85)	2.43 (2.06)	2.35 (1.93)	[2.12–2.64]
PMC ^2^								
Object control	3.98 (0.88)	3.75 (0.78)	3.88 (0.85)	[3.78–3.99]	3.86 (0.87)	3.63 (0.87)	3.77 (0.88)	[3.69–3.93]
Self-movement	3.40 (0.95)	3.83 (0.86)	3.58 (0.93)	[3.49–3.73]	3.15 (0.93)	3.39 (0.87)	3.25 (0.91)	[3.16–3.40]

^1^ Actual motor competence: range from 0 to 8. ^2^ Perceived motor competence: range from 1 to 5. CI: confidence interval.

## Data Availability

The underlying research materials related to this paper are available from the corresponding author upon request.
